# Neurosteroid Metabolites of Gonadal Steroid Hormones in Neuroprotection: Implications for Sex Differences in Neurodegenerative Disease

**DOI:** 10.3389/fnmol.2018.00359

**Published:** 2018-10-05

**Authors:** Ari Loren Mendell, Neil James MacLusky

**Affiliations:** Department of Biomedical Sciences, Ontario Veterinary College, University of Guelph, Guelph, ON, Canada

**Keywords:** neurosteroids, sex differences, Alzheimer’s disease, neuroprotection, neurodegenerative disease

## Abstract

Gonadal steroid hormones are neurotrophic and neuroprotective. These effects are modulated by local metabolism of the hormones within the brain. Such control is necessary to maintain normal function, as several signaling pathways that are activated by gonadal steroid hormones in the brain can also become dysregulated in disease. Metabolites of the gonadal steroid hormones—particularly 3α-hydroxy, 5α-reduced neurosteroids—are synthesized in the brain and can act through different mechanisms from their parent steroids. These metabolites may provide a mechanism for modulating the responses to their precursor hormones, thereby providing a regulatory influence on cellular responses. In addition, there is evidence that the 3α-hydroxy, 5α-reduced neurosteroids are neuroprotective in their own right, and therefore may contribute to the overall protection conferred by their precursors. In this review article, the rapidly growing body of evidence supporting a neuroprotective role for this class of neurosteroids will be considered, including a discussion of potential mechanisms that may be involved. In addition, we explore the hypothesis that differences between males and females in local neurosteroid production may contribute to sex differences in the development of neurodegenerative disease.

## Introduction

Many neurodegenerative diseases—conditions that are characterized by progressive deterioration of cognition, mood, executive function, etc. due to loss of neurons in the central nervous system (CNS)—exhibit sex differences in incidence, onset and severity (for review see Hanamsagar and Bilbo, 2016). For example, while most studies indicate that men are more likely to develop amyotrophic lateral sclerosis (ALS; McCombe and Henderson, 2010) and Parkinson’s disease (Baldereschi et al., 2000; Elbaz et al., 2002), the opposite is true for the development of Alzheimer’s disease (AD; Seshadri et al., 1997; Plassman et al., 2011; Alzheimer’s Association, 2014) and multiple sclerosis (MS; Voskuhl and Gold, 2012), with women comprising approximately two-thirds of patients living with both conditions (Hebert et al., 2013; Hanamsagar and Bilbo, 2016). Several potential causes for sex differences in neurodegenerative disease have been proposed over the years, including interactions between genetic background and environment (for review, Xu et al., 2015), as well as sex differences in life expectancy and incidence of non-neurodegenerative conditions with high mortality rates (Alzheimer’s Association, 2014).

One of the most prominent hypotheses for sex differences in the development of some of these conditions is the profound difference in the timing, extent and duration of decline in the levels of circulating gonadal steroid hormones. In women, the relatively abrupt and drastic menopausal decline in circulating ovarian steroids has been suggested to increase the risk for the development of AD, with a corresponding reduction in risk in estrogen replacement therapy users (Paganini-Hill and Henderson, 1994, 1996; Tang et al., 1996; Kawas et al., 1997), although the relationship is complex because other studies have also shown a positive correlation between estrogen exposure and risk of dementia (Geerlings et al., 2001; Schumacher et al., 2003). This relationship has been further complicated by equivocal findings regarding the role of progesterone on risk of dementia, specifically with respect to its interactions with estrogen in combination therapies (Shumaker et al., 2003; Honjo et al., 2005), with limited investigation of the effects of progesterone independent of estrogen. In men, the natural age-related decline in circulating testosterone levels is much more gradual than the menopausal decline in ovarian steroid hormones observed in women. Declining testosterone levels have been associated with an increased risk for the development of AD and cognitive decline (Hogervorst et al., 2001, 2003, 2004; Yeap et al., 2008; Hsu et al., 2015). Because of these findings, estrogen and testosterone are believed to be neuroprotective in women and men, respectively (for review, see Pike, 2017).

While traditional views of gonadal steroid hormone actions on the brain were predicated on the concept that circulating hormones were required to cross the blood-brain-barrier in order to exert their effects, the discovery of neurosteroids—steroid hormones that are synthesized *de novo* within the nervous system—has broadened the scope of the potential for steroid hormones to impact structure, activity and function of neurons and other cells throughout the CNS. The enzymes necessary for conversion of the primary gonadal steroid hormones to other biologically active metabolites are present in the brain. This raises the possibility that gonadal steroid hormone-mediated neuroprotection, and sex differences in the development of neurodegenerative diseases, may be impacted by neurosteroids that act through distinct mechanisms from their precursors.

In recent years, this hypothesis has gained added impetus from the recognition that the neuroprotective effects of steroids, whether synthesized locally or derived from peripheral steroidogenesis, may be augmented and diversified through local conversion to metabolites with biological properties different from those of their parent hormones. This is a very large and rapidly growing field, with elements beyond the scope of the present article to adequately review. For further information in this area, the reader is referred to a number of excellent recent reviews on the neuroprotective effects of gonadal steroids (Galea et al., 2017; Pike, 2017; Choleris et al., 2018; Giatti et al., 2018). The present review focuses more specifically on the 3α-hydroxy, 5α-reduced metabolites of circulating and neuro-steroids, as well as potential mechanisms through which they may exert neuroprotective effects in the nervous system. In addition, we explore the hypothesis that sex differences in the development of neurodegenerative disease may be partially attributable to differences in neurosteroidogenesis between males and females.

## Neurosteroids

The study of neurosteroids has expanded enormously since the first demonstration that the key enzyme required for the biosynthesis of steroid hormones, the cytochrome P-450 cholesterol side-chain cleavage enzyme (P450scc) was widely distributed throughout the brain (Baulieu and Robel, 1990; Baulieu, 1997; Baulieu et al., 2001). These initial findings suggested that the biosynthetic pathways necessary to synthesize steroid hormones might be expressed within the nervous system. Steroidogenic enzymes including the P450scc, 17α-hydroxylase, 21-hydroxylase, P450 aromatase, 17β-hydroxysteroid dehydrogenase (17β-HSD), 5α-reductase, 3α-hydroxysteroid dehydrogenase (3α-HSD) and 3β-hydroxysteroid dehydrogenase (3β-HSD), are present in many different areas of the brain (Baulieu and Robel, 1990; Melcangi et al., 1996; Mellon and Griffin, 2002; Taves et al., 2011). The diversity of expression of these enzymes in the nervous system allows for the production of the principal steroid hormones, including cortisol (or corticosterone in rodents), progesterone, estradiol and testosterone. This local neurosteroidogenesis, combined with systemically-derived steroids that cross the blood-brain barrier, contribute to the importance of steroid hormones as modulators of neuronal function and survival. These hormones exert sex-specific effects on neural cells, as steroid receptors are differentially expressed throughout the brain in males and females of non-human primates and rodents (Finley and Kritzer, 1999; Milner et al., 2001, 2005; Adams et al., 2002; Nuñez et al., 2003; Tabori et al., 2005; Kritzer, 2006; Sarkey et al., 2008; Wang A. C. J. et al., 2010; Duarte-Guterman et al., 2015; Mogi et al., 2015). However, the influence of these steroid hormones on neural development, activity and survival are not limited to effects mediated via their respective steroid receptor systems. Over the past three decades, the actions of neurosteroid metabolites of gonadal steroid hormones that are synthesized in relatively high concentrations in the brain have received a great deal of attention, due to their ability to act through mechanisms that both converge and diverge from their parent hormones. Of particular interest has been their ability to modulate the activity and sensitivity of specific neurotransmitter receptors, but additional mechanisms by which neurosteroids may affect the function of cells in the brain have also been identified.

## 3α-Hydroxy, 5α-Reduced Neurosteroids as Regulators of GABA_A_ Receptor Activity

**Figure 1:**
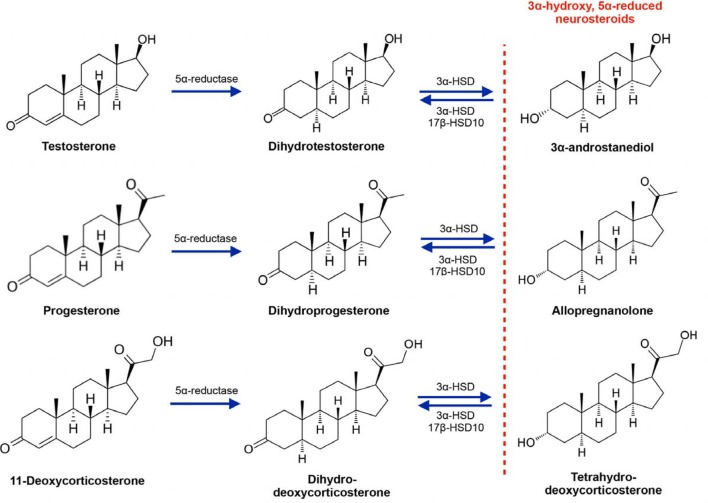
Synthesis of the 3α-hydroxy, 5α-reduced neurosteroid metabolites of testosterone, progesterone and 11-deoxycorticosterone. 3α-HSD, 3α-hydroxysteroid dehydrogenase; 17β-HSD10, 17β-hydroxysteroid dehydrogenase (17β-HSD) type 10.

Neurosteroid metabolites of gonadal steroid hormones can modulate the effects of neurotransmitters at their receptors, especially the inhibitory neurotransmitter γ-Aminobutyric acid (GABA; Reddy et al., 2004; Belelli and Lambert, 2005; Reddy, 2008; Reddy and Jian, 2010; Carver and Reddy, 2013). Several neurosteroids are able to allosterically modulate the GABA_A_ receptor, which is a pentamer of subunits with a chloride ion channel at the core. Neurosteroids with 3α-hydroxy, 5α-reduced structures are positive allosteric modulators of GABA_A_ receptor activity. This includes metabolites of testosterone, progesterone and 11-deoxycorticosterone that have been sequentially reduced by 5α-reductase and 3α-HSD (most notably 3α-HSD type 3 in the brain) to produce 5α-androstane-3α, 17β-diol (3α-diol), 5α-pregnane-3α-ol-20-one (allopregnanolone) and 3α,21-dihydroxy-5α-pregan-20-one (tetrahydrodeoxycorticosterone, THDOC; Figure 1). The 3α-hydroxy, 5α-reduced structure is essential for allosteric modulation of GABA_A_ activity, as 3β analogs (produced through sequential reduction by 5α-reductase and 3β-HSD) either have negative or null activity at the GABA_A_ receptor. Importantly, the affinity and relative potency of 3α-hydroxy, 5α-reduced for the GABA_A_ receptor is highly dependent on the subunit composition of the receptor itself. The GABA_A_ receptor is a pentamer that is typically composed of two α subunits (α1–6), two β subunits (β1–4), and one additional subunit that is either a γ (γ1–3), δ, ε, π, or θ, or ρ (ρ1–3; Sieghart, 2006; Reddy and Jian, 2010; Belelli et al., 2018). A low-affinity human β3 homopentamer of the GABA_A_ has been identified as well (Miller and Aricescu, 2014), though this conformation of the receptor does not appear to be neurosteroid-sensitive, probably due to the lack of α subunits (Nik et al., 2017). The subunit composition of the receptor differs depending on its location on the neuronal membrane. For example, receptors containing α1 and γ2 subunits are usually located within synapses, while receptors containing α4/α6 and δ subunits are extrasynaptic, generally being present on or near the cell body of neurons. While most heteropentamers are sensitive to modulation by neurosteroids, extrasynaptic receptors containing δ subunits have been shown to have relatively greater sensitivity. Neurosteroids bind to a distinct site within the core of the ion channel at α- and β-subunit transmembrane domains, whereas GABA itself binds to a site that is between α and β subunits (Hosie et al., 2006).

While the effects of 3α-hydroxy, 5α-reduced neurosteroids via regulation of GABA_A_ receptor activity are relatively well characterized, other mechanisms have also been proposed. Some identified targets of allopregnanolone include the pregnane-X-receptor (PXR), liver-X-receptor (LXR), membrane progesterone receptor (mPR), N-Methyl-D-Aspartate (NMDA) receptor and L- or T-type calcium channels (Wang et al., 2005; Chen et al., 2011; Frye and Paris, 2011), while the testosterone metabolite 3α-diol has been shown to have negligible affinity for the androgen receptor (AR) and weak affinity for estrogen receptor β (ERβ; Liao et al., 1973; Kuiper et al., 1997; Penning, 1997; Edinger and Frye, 2007). Further work seems warranted in order to continue to identify and characterize mechanisms of action for these neurosteroids.

Because of their ability to modulate multiple signaling pathways, 3α-hydroxy, 5α-reduced neurosteroids have been shown to play important roles in physiological and pathophysiological regulation of stress, mood and feeding behavior (reviewed in Gunn et al., 2011; Mody and Maguire, 2012; Bäckström et al., 2014; Holmberg et al., 2018). In addition, and of particular interest for the focus of this review, 3α-hydroxy, 5α-reduced neurosteroids have been shown to have therapeutic potential for use in treatment of neurodegenerative and neurological diseases.

## Allopregnanolone in Neuroprotection

### Allopregnanolone in Neuropathic Pain

Neuropathy is a persistent issue in patients undergoing chemotherapy, as well as a common symptom in patients with diabetes. Allopregnanolone has been widely shown to protect against physiological, biochemical and functional alterations associated with peripheral neuropathy. Anti-cancer drugs including Oxaliplatin and Vincristine have been shown to induce alterations to the normal function of peripheral nerves, decrease thermal and mechanical pain thresholds, and reduce sciatic nerve conduction velocity and action potential peak amplitude in adult male Sprague-Dawley rats (Meyer et al., 2010, 2011). In addition, protein markers for myelination of peripheral axons in the sciatic nerve have been found to be decreased by these treatments (Meyer et al., 2010). These effects were restored to normal by treatment with progesterone, 5α-dihydroprogesterone (DHP), or allopregnanolone (Meyer et al., 2010, 2011), with the precursors conceivably achieving these effects at least in part through conversion to allopregnanolone. Allopregnanolone has also been shown to protect against molecular and functional aspects of neuropathic pain induced by median nerve chronic constriction injury (CCI) in male Sprague-Dawley rats (Huang et al., 2016). In this study, treatment with allopregnanolone inhibited astrocytic and microglial ERK phosphorylation in the cuneate nucleus, concurrent with a reduction in pain hypersensitivity. This was also associated with a reduction in glial activation and pro-inflammatory cytokine expression in the cuneate nucleus. The observed protection was apparently exerted through potentiation of GABA_A_ receptor activity, as bicuculline blocked the effects of allopregnanolone. These effects were proposed to be independent of reverse metabolism to DHP, as the synthetic progestin and allopregnanolone synthesis inhibitor, medroxyprogesterone, reduced allopregnanolone levels in the cuneate nucleus and exacerbated neuropathic pain parameters induced by CCI (Huang et al., 2016). However, it should be noted that the formulation of medroxyprogesterone (medroxyprogesterone acetate) used in the study discussed above has also been shown to impact the activation of other steroid hormone receptors, particularly androgen and glucocorticoid receptors (Stanczyk et al., 2013; Africander et al., 2014), which could have contributed to its effects.

Chronic streptozotocin exposure is a well-established method for induction of type 1 diabetes in rodent models. This provides a valuable model to study the effects of diabetes on peripheral neuropathy. Using this model in male Sprague-Dawley rats, Leonelli et al. (2007) demonstrated that plasma progesterone levels are reduced in diabetic mice, and chronic treatment with progesterone, DHP, or allopregnanolone were effective in differentially restoring their circulating levels and counteracting impaired nerve conduction velocity of peripheral nerves. This effect was accompanied by an increase in thermal pain threshold and skin innervation density. In this study, levels of myelin protein markers and Na^+^/K^+^-ATPase activity were increased by progesterone and DHP, but not allopregnanolone (Leonelli et al., 2007).

In addition to peripheral neuropathy, allopregnanolone has been shown to alleviate some deficits in a mouse model of human immunodeficiency virus (HIV) neuropathy. The trans-activator of transcription (Tat) is a neurotoxic protein associated with neurological deficits in HIV patients. By using a mouse model that conditionally expressed Tat, Paris et al. (2016) were able to assess the impact of progesterone and its 5α-reduced metabolites on various neurological and pathophysiological deficits induced by Tat. They found that Tat expression in ovariectomized female mice resulted in an increase in anxiety-like behavior, and this was attenuated by progesterone. However co-treatment with finasteride (a 5α-reductase inhibitor) abolished the progesterone-mediated protection, suggesting that the protective effects were mediated by neurosteroid metabolites as opposed to progesterone itself. In the same study, allopregnanolone partially protected striatal neuronal-glial co-cultures against neurotoxicity and intracellular calcium accumulation induced by Tat treatment.

### Allopregnanolone in Amyotrophic Lateral Sclerosis (ALS) and Multiple Sclerosis (MS)

Allopregnanolone is a promising candidate for the treatment of conditions that present with both neuroinflammation and neurodegeneration, as it has been shown to protect against the molecular and neurotoxic consequences of both processes. Two disorders that include both inflammatory and degenerative components are ALS and MS. As such, several studies have been performed in rodent models of these conditions in order to characterize protective effects of allopregnanolone. Deniselle et al. (2012) used Wobbler mice—a model for sporadic ALS—in order to evaluate the effects of chronic progesterone treatment on mitochondrial function. These mice had reductions of mitochondrial complex I and II-III activity, as well as increased levels of mitochondrial neuronal nitric oxide synthase (nNOS), which leads to toxic levels of nitric oxide and results in oxidative stress in the spinal motoneurons of these mice. Progesterone improved mitochondrial complex I activity in the cervical region of the spinal cord, reduced mitochondrial nNOS levels and prevented alterations in amyloid precursor protein (APP) and MnSOD levels in motoneurons (Deniselle et al., 2012). In a follow up study, the same researchers demonstrated that allopregnanolone was able to produce similar protective effects in the Wobbler mice as progesterone had in the previous study. Both steroids were able to attenuate dysregulations in Akt and c-Jun N-terminal kinase (JNK) phosphorylation, motoneuron vacuolation, BDNF/TrkB mRNA expression, choline acetyltransferase (ChAT) and forelimb grip strength (Meyer et al., 2017). Another study by the same group found that the synthetic progestin Nestorone exerted many of the same beneficial effects in male Wobbler mice as progesterone and allopregnanolone (Meyer et al., 2015). In contrast, however, the effects appeared to be primarily dependent on progesterone receptor signaling, as conversion of Nestorone to its 3α-hydroxy, 5α-reduced was limited, and this metabolite did not possess substantial modulatory activity of the GABA_A_ receptor (Kumar et al., 2017). Therefore, while allopregnanolone is important for progesterone-mediated neuroprotection in the Wobbler mouse, it does not appear to account for all of its beneficial effects.

One of the earliest studies establishing that neurosteroids may modulate factors related to myelination demonstrated that allopregnanolone treatment stimulated peripheral myelin protein 22 (P22) and myelin protein 0 (P0) gene expression in adult male rats, and in Schwann cell cultures (Melcangi et al., 1999). Based on their findings, it was therefore suggested that endogenous allopregnanolone may promote the expression of myelin proteins within the peripheral nervous system (Melcangi et al., 1999). Demyelination is a major pathophysiological characteristic of disorders with neuroinflammatory components, such as MS, and potential roles for neurosteroids on myelination in the CNS have been investigated. Several studies used the common experimental autoimmune encephalomyelitis (EAE) mice to investigate whether exogenous progesterone treatment could reduce the development of MS-related pathology, with the effects possibly being driven through conversion to allopregnanolone. In male EAE mice, it was demonstrated that administration of a progesterone pellet 1 week prior to EAE induction reduced demyelination, markers of axonal pathology and neuronal dysfunction/degeneration in spinal cord motoneurons (Garay et al., 2007, 2009). Interestingly, in the same EAE animal model, progesterone has recently been shown to modulate the expression of a number of mRNAs coding for enzymes involved in steroidogenesis, suggesting that in addition to acting as a substrate for neurosteroid production, progesterone may itself alter local steroidogenesis in the spinal cord (Garay et al., 2017).

### Allopregnanolone in Parkinson’s Disease

Parkinson’s disease is a neurodegenerative condition with components involving degeneration of areas related to motor control, coordination and cognitive function. The actions of neurosteroids are known to be altered in Parkinson’s patients, and this has been related to altered expression of biosynthetic enzymes and receptor subunit targets of neurosteroids. In the substantia nigra (SN) of Parkinson’s disease patients, 5α-reductase type 1 and GABA_A_ receptor subunits α4 and β1 were found to be downregulated, while 3α-HSD type 3 and GABA_A_ receptor subunit α4 were upregulated in the cuneate nucleus (Luchetti et al., 2010). The latter could potentially be a compensatory response, in order to increase the local synthesis of 3α-hydroxy, 5α-reduced neurosteroids such as allopregnanolone and 3α-diol, along with one of their receptor subunit targets that is more sensitive to these metabolites (Carver and Reddy, 2013).

Studies in animal models have further characterized protective mechanisms of allopregnanolone in Parkinson’s disease. The widely employed 1-methyl-4-phenyl-1,2,3,6-tetrahydropyridine (MPTP) lesion mouse model of Parkinson’s disease (Meredith and Rademacher, 2011) was used to evaluate protective effects of allopregnanolone. In this study, it was found that allopregnanolone increased tyrosine hydroxylase (TH)-positive cells and total neurons in the SN pars compacta (SNpc) and the locus coeruleus (LC), and increased norepinephrine levels in the striatum of MPTP-lesioned male C57Bl/6J mice (Adeosun et al., 2012). In addition, allopregnanolone increased BrdU^+^ neurons in the SNpc and improved performance of MPTP-lesioned mice on the rotarod test of balance and coordination (Adeosun et al., 2012).

### Allopregnanolone in Ischemia and Traumatic Brain Injury (TBI)

Many peripheral and central conditions can result in cerebral ischemia, and primary gonadal steroids (particularly progesterone) have been shown to have promise in protection during the recovery from ischemic insult (for review see Sayeed and Stein, 2009). Because of the central conversion of progesterone to allopregnanolone, studies have also investigated a potential role for neurosteroid metabolites of gonadal steroid hormones in the protection of neural cells following ischemic insult and reperfusion.

Cardiac arrest is known to result in neuronal cell death as a result of global cerebral ischemia. Some areas of the brain are more susceptible to detrimental consequences of this ischemia than others, including the striatum, cerebellum and CA1 hippocampal subfield. In mice, the cardiac arrest requiring cardiopulmonary resuscitation (CA/CPR) model has been shown to recapitulate many of the effects seen in humans following such an event. In studies investigating cells in one of these vulnerable areas—Purkinje cells of the cerebellum—it was demonstrated that CA/CPR reduced GABA_A_ receptor expression induced Purkinje cell death, while allopregnanolone was effective in preventing or reversing these consequences (Kelley et al., 2008, 2011). Interestingly, while Purkinje cell death was reduced by both 2 mg/kg and 8 mg/kg doses in adult female C57bl/6 mice, males were only protected by the higher dose (Kelley et al., 2011). Allopregnanolone increased inhibitory neurotransmission in both sexes, but was more effective in females (Kelley et al., 2011), further illustrating the sex difference in susceptibility. Similar experiments have confirmed these effects of allopregnanolone *in vitro*, demonstrating that cultured cerebellar Purkinje cells treated with allopregnanolone had recovered synaptic and total GABA_A_ receptor current and increased α1 subunit expression that were reduced by oxygen glucose deprivation (OGD; Kelley et al., 2008). These effects were consistent with an earlier report that demonstrated dose-dependent neuroprotection of cerebellar Purkinje cells by progesterone treatment following OGD, acting through a mechanism that was dependent on conversion to allopregnanolone (Ardeshiri et al., 2006). In that particular study, cultures from male mice required higher concentrations of progesterone to reduce cell death, consistent with the reports *in vivo* (Ardeshiri et al., 2006).

As opposed to the global ischemia induced by CA/CPR, middle cerebral artery occlusion (MCAO) can induce focal cerebral ischemia, which provides a model of ischemic stroke. Allopregnanolone and progesterone have been shown to significantly reduce infarct volume in several brain areas following MCAO in male Sprague-Dawley rats, including in the cerebral cortex (Sayeed et al., 2006, 2007). Interestingly, cortical infarct volume was found to be effectively reduced by a lower dose of allopregnanolone compared to progesterone (Sayeed et al., 2006), suggesting that progesterone may actually exert its effects through a mechanism that is at least partially dependent on conversion to allopregnanolone. Progesterone treatment was also shown to improve rotorod performance in rats following MCAO, indicating a potential role in improving balance and motor coordination during stroke recovery (Sayeed et al., 2007).

Traumatic brain injury (TBI) has aspects of pathology related to both direct damage to neurons from impact, as well as prolonged damage to ischemia or vessels and cells responsible for oxygenation and support of neurons. Using a controlled frontal cortical contusion model of TBI, it has been shown that allopregnanolone reduces the degree of apoptosis and cell loss, learning and memory deficits, and astrocyte infiltration to the site of injury, without affecting the size of the cavity induced by injury itself (Djebaili et al., 2004, 2005). This effect was also achieved with progesterone treatment, but consistent with the studies investigating the two steroid hormones on the effects of ischemia described above, allopregnanolone was effective at lower doses (Djebaili et al., 2004, 2005).

### Allopregnanolone in Alzheimer’s Disease

While allopregnanolone has been widely studied for both its endogenous role in the development and prevention of neurological and neurodegenerative diseases, as well as its potential use as an exogenous therapeutic, no condition has received more attention with respect to the potential protective applications of allopregnanolone than AD. Over the past two decades, studies from several research groups have investigated the mechanisms by which allopregnanolone may act as a protective and regenerative therapeutic in this disease.

One of allopregnanolone’s most intriguing properties, in terms of its potential application to treatment of AD, is its ability to enhance proliferation of neural progenitor cells (NPCs) to create new neurons in the hippocampus. In male triple transgenic AD (3xTg-AD) mice, it has been consistently shown that allopregnanolone increases neurogenesis and promotes the survival of newly generated neural cells (Wang J. M. et al., 2010; Chen et al., 2011; Singh et al., 2012). In addition, allopregnanolone can reduce Aβ pathology in the hippocampus, cortex, and amygdala of male 3xTg-AD mice (Chen et al., 2011), and improve hippocampal-dependent associative learning and memory (Wang J. M. et al., 2010; Singh et al., 2012). An important consideration is that allopregnanolone was most effective if administered prior to the deposition of extraneuronal Aβ plaques (Chen et al., 2011; Singh et al., 2012). In addition, allopregnanolone can reduce the infiltration and activation of microglia and increase myelination of oligodendrocytes (Chen et al., 2011), consistent with reports from rodent models of other conditions (for details, see the neuropathy and ALS/MS sections above). Systems that regulate neurotransmitter levels may able be targets for protection by allopregnanolone. In the SNpc of young 3xTg-AD male mice, TH and total neuron levels were increased to control levels by a single allopregnanolone injection (Sun et al., 2012). Similar findings have been reported in young APPswe/PSEN1 double transgenic male mice, where these changes were accompanied by an increase in proliferation and survival of new neurons in the subventricular zone, and increased neuronal survival in the SNpc (Zhang et al., 2015).

Allopregnanolone promotes proliferation of NPCs in the subgranular zone (SGZ) of the dentate gyrus due to the unique nature of the GABAergic activation of cells in this area (Irwin et al., 2012). The NPCs in the SGZ retain a state similar to that of embryonic neurons, in which the GABA_A_ receptor populations trigger an efflux of chloride upon opening, thereby depolarizing the cell (Tozuka et al., 2005; Irwin et al., 2012). Additionally, the predominant GABA_A_ receptor subunits expressed on these cells is consistent with highly neurosteroid-sensitive, extrasynaptic GABA_A_ receptors (containing α subunits that are often found with δ subunit-containing receptors; Stell et al., 2003; Overstreet Wadiche et al., 2005; Irwin et al., 2012). The depolarization caused by the efflux of chloride upon GABA_A_ receptor channel opening results in the activation of voltage-dependent L-type calcium channels (Wang et al., 2005). This activates calcium-dependent kinases including the protein kinase A (PKA)/cyclic AMP (cAMP)/cAMP response element binding protein (CREB) pathway, leading to the upregulation of pro-mitotic and suppression of anti-mitotic gene expression (Wang and Brinton, 2008; Irwin et al., 2012). Interestingly, allopregnanolone has also been shown to increase intracellular calcium at low concentrations (EC50 of 10 ± 4 nM) in fetal hypothalamic neurons (Dayanithi and Tapia-Arancibia, 1996). The GABA_A_ receptor-mediated effects are stereoselective, as 3β stereoisomers and steroids that have a removal, addition or alteration of hydroxyl functional groups, abolishing the effects at the GABA_A_ receptor, fail to display the induction of proliferation in various *in vitro* and *in vivo* models (Brinton, 1994; Wang et al., 2005; Wang and Brinton, 2008; Karout et al., 2016).

Recent studies have also demonstrated the potential involvement of other mechanisms by which allopregnanolone may protect neural cells independent of proliferation of NPCs. Allopregnanolone has been shown to increase the expression of proteins that promote the clearance and homeostasis of cholesterol, including the LXR, the PXR and methyl-glutaryl-CoA-reductase (HMG-CoA-R; Chen et al., 2011). Interestingly, while the 3α-hydroxy conformation appears to be required to stimulate proliferation, 3β isomers of allopregnanolone and synthetic analogs (3-O-allyl-allopregnanolone) have been shown to protect hippocampal neurons, neural stem cell cultures, and SH-SY5Y human female neuroblastoma cells from neurotoxicity induced by amyloid β peptide 1-42 (Aβ42) or hydrogen peroxide (H_2_O_2_; Karout et al., 2016; Lejri et al., 2017). The mechanisms of allopregnanolone and its analogs on neuroprotection *in vitro* appear to have both GABA_A_ receptor-dependent and -independent components, as these neurosteroids are able to exert protective effects against Aβ42 or H_2_O_2_-induced toxicity in the absence of functional GABA_A_ receptors (Lejri et al., 2017; Mendell et al., 2018); although allopregnanolone appears to be more effective at lower concentrations in the presence of functional GABA_A_ receptors (Grimm et al., 2014; Karout et al., 2016; Mendell et al., 2018). Further studies are required in order to better characterize the GABA_A_ receptor-independent mechanisms by which allopregnanolone may provide neuroprotection.

## Testosterone Metabolism in Neuroprotection

While the majority of studies investigating the potential protective effects of 3α-hydroxy, 5α-reduced neurosteroids have focused on allopregnanolone, other delta-4 3-keto hormonal steroids can also be converted to analogous bioactive metabolites. Recent work has expanded studies to the testosterone-derived structural analog of allopregnanolone, 3α-diol. Several studies have demonstrated that 3α-diol is effective in reducing peripheral neuropathy resulting from different chemical and physical insults (Meyer et al., 2013). Either prophylactic or corrective 3α-diol was shown to suppress paclitaxel-induced thermal and mechanical pain sensitivity, while restoring nerve conduction velocity and action potential peak amplitude in the sciatic nerve, as well as intraepidermal nerve fiber density in male Sprague-Dawley rats (Meyer et al., 2013). There was also evidence that 3α-diol repaired nerve damage in peripheral axons (Meyer et al., 2013). In another study investigating neuropathic pain related to induction of diabetes using streptozotocin injection, either DHT or 3α-diol had analgesic properties in diabetic male Sprague-Dawley rats (Calabrese et al., 2014). Interestingly, the mechanisms by which they improved neuropathic pain had both convergent and divergent components. Both neurosteroids reduced glutamate release, astrocyte infiltration and IL-1β expression. However, DHT increased mechanical nociceptive threshold and reduced over-activation of synapsin-1, syntaxin-1 and P-GluN2B, while 3α-diol increased tactile allodynia threshold and reduced mRNA expression of substance P, toll-like receptor 4, tumor necrosis factor-α, and TSPO (Calabrese et al., 2014). In an earlier study by Magnaghi et al. (2004), 3α-diol was also shown to promote the expression of factors related to peripheral myelination. In male Sprague-Dawley rats, gonadectomy decreased the expression of P0 and P22—two proteins that play a crucial physiological role in the maintenance of the multilamellar structure of peripheral myelin—and the levels of these proteins were recovered by treatment with 3α-diol, while DHT only recovered P0 expression (Magnaghi et al., 2004).

Many studies have evaluated cognitive outcomes after gonadectomy in male animals (for review, see Leonard and Winsauer, 2011). However, a limited number of these studies have evaluated the potential role of 3α-diol in effects that are largely attributed to testosterone, and loss thereof following gonadectomy. In a study using a battery of behavioral tests to evaluate the effects of gonadectomy and replacement with testosterone or 3α-diol in male Fisher-344 rats, Frye et al. (2010) found that age-related decline in performance on cognitive and affective tasks was associated with a decrease in hippocampal 3α-diol levels. Treatment with 3α-diol, but not testosterone, was able to reverse age-related decline in performance. Similar declines in performance were observed after gonadectomy in younger animals, and these were reversed by long-term testosterone replacement using Silastic capsules, which were found to increase both testosterone and 3α-diol levels in the hippocampus (Frye et al., 2010). In another study by the same group, gonadectomized young adult male Long-Evans rats were treated with either testosterone, DHT, or 3α-diol by long-term Silastic capsule implant or acute intrahippocampal infusion (Edinger and Frye, 2004). Animals treated with any of the steroids displayed improved measures of analgesia, less anxiety-like behavior, and increased learning compared to control animals. However, only hippocampal 3α-diol levels were positively correlated with performance on the various tasks, suggesting that effects were primarily mediated through 3α-diol as opposed the androgenic precursors (Edinger and Frye, 2004).

### Evidence for a Protective Role of 3α-diol in Alzheimer’s Disease

Several *in vitro* studies performed by our group and others have demonstrated that 3α-diol may play an important role in protecting against various molecular and cellular dysfunctions associated with the various pathophysiological processes that occur in the brains of individuals with AD.

**Figure 2:**
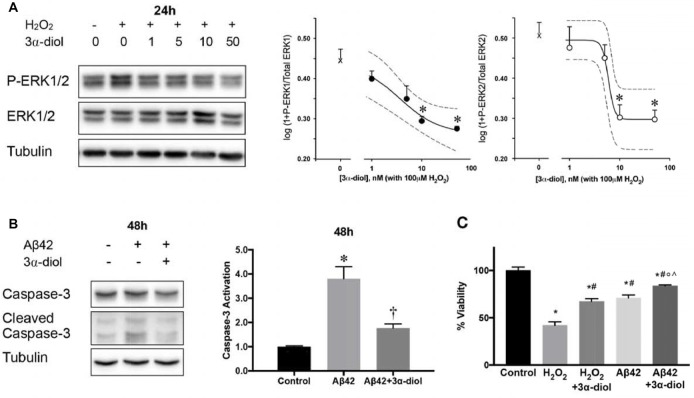
3α-androstanediol (3α-diol) inhibits ERK phosphorylation induced by hydrogen peroxide (H_2_O_2_) in a dose-dependent manner in SH-SY5Y cells **(A)**. 3α-diol reduces apoptosis induced by amyloid Beta peptide 1–42 (Aβ42), as indicated by decreased caspase-3 activation **(B)**, and dampens the loss in cellular viability induced by either H_2_O_2_ or Aβ42 **(C)**. For panel **(A)**, * represents *p* < 0.05 compared to the H_2_O_2_ group. For panel **(B)**, * and † represent *p* < 0.05 compared to the control and Aβ42 groups, respectively. For panel **(C)**, *, #, ◦, and ∧ represent *p* < 0.05 vs. the control, H_2_O_2_, H_2_O_2_ + 3α-diol, and Aβ42 groups, respectively. This figure is a compilation of illustrations from Mendell et al. (2016); reproduced by permission of Oxford University Press.

Physiological concentrations of 3α-diol are able to protect SH-SY5Y neuroblastoma cells and primary cortical neurons isolated individually from male and female mice against neurotoxicity induced by H_2_O_2_ or Aβ42 (Mendell et al., 2016, 2018; Figure 2). This protection appeared to occur through 3α-diol-mediated inhibition of prolonged ERK phosphorylation induced by the neurotoxins, and was associated with a reduction in caspase-3 activation and cell death (Mendell et al., 2016; Figure 2). Interestingly, these effects appeared to be GABA_A_ receptor-independent, as they were essentially the same in the absence (SH-SY5Y cells) or presence (primary cortical neurons) of functional neurosteroid-sensitive GABA_A_ receptors, and were not prevented by picrotoxin (Mendell et al., 2018). Allopregnanolone was found to exert effects in both *in vitro* models, but required a 10-fold higher concentration than 3α-diol in the absence of GABA_A_ receptors, while the effects on ERK phosphorylation were largely prevented by picrotoxin in cells expressing GABA_A_ receptors (Mendell et al., 2018). These results are consistent with other findings on neurosteroid mediation of mitochondrial bioenergetics, which demonstrated that 10 nM concentrations of 3α-diol, but not allopregnanolone, were effective in improving aspects of mitochondrial function in GABA_A_ receptor deficient SH-SY5Y cells, although both steroids were effective in primary cortical neurons (Grimm et al., 2014). Experiments designed with the aim of identifying the GABA_A_ receptor-independent mechanisms of 3α-diol action are ongoing, but as discussed above for allopregnanolone, further work will be necessary to characterize these effects.

These observations suggest a potentially important difference between males and females in the role of neurosteroidogenesis in the development of AD. In both men and women with AD, allopregnanolone has been found to decline in circulation and in various brain regions compared to normal control subjects (Bernardi et al., 2000; Yang and He, 2001; Marx et al., 2006; Smith et al., 2006; Naylor et al., 2010). By contrast, the available evidence suggests that neurotoxicity, including neurotoxicity associated with exposure to Aβ, may not reduce 3α-diol levels. In SH-SY5Y human neuroblastoma cells, 3α-diol is not reduced following H_2_O_2_-induced oxidative stress or exogenous Aβ exposure (Schaeffer et al., 2006, 2008), but increases in cells over-expressing APP (Schaeffer et al., 2006). *In vivo*, limbic brain concentrations of 3α-diol increase with age in wild-type and 3xTg-AD male mice (Caruso et al., 2013a). Consistent with these results, a divergence between plasma levels of allopregnanolone and 3α-diol has been reported in patients with AD, the former being significantly reduced in patients with the disease, while the latter does not appear to change, compared to normal age-matched controls (Smith et al., 2006). Taken together, these observations suggest that differences in the relative production rates of neurosteroids could affect susceptibility to AD. Brain concentrations of allopregnanolone in individuals with AD decline in both sexes, but there is relatively little change (and possibly even a compensatory increase) in brain 3α-diol levels. Higher levels of free androgen in the circulation of males could exacerbate the situation, by providing higher concentrations of testosterone as a substrate for local 3α-diol biosynthesis. If so, females could be at higher risk for disease development as a result of a relatively greater net loss of 3α-hydroxy, 5α-reduced neurosteroids with age. While this hypothesis remains speculative, because there is as yet insufficient direct comparative data on brain levels of different neurosteroids in males and females during both normal and pathological aging, further study of 3α-diol levels in relation to sex differences in the onset and progress of AD seems warranted.

## TSPO Ligands in Neuroprotection

The 18 kDa translocator protein (TSPO) is believed to be an important regulator of steroid synthesis by enhancing the translocation of cholesterol into the mitochondria (Papadopoulos et al., 2006; Fan et al., 2015). Along with the steroidogenic acute regulatory protein (StAR), TSPO has been hypothesized to be essential to neurosteroid synthesis (Papadopoulos et al., 2006; Rupprecht et al., 2010), though there have recently been challenges to its necessity for adrenal and gonadal steroidogenesis using conditional TSPO knockout models (Morohaku et al., 2014; Fan et al., 2015). Because of its role in steroidogenesis, TSPO has been increasingly studied in recent years as a potential therapeutic target in disorders that have shown improvement in preclinical models following neurosteroid manipulation (Rupprecht et al., 2010). Pharmaceutical compounds that are known to act as ligands for TSPO have been shown to induce neurosteroidogenesis, and this has been utilized to determine whether enhanced neurosteroidogenesis may be beneficial in several different models of neurodegenerative and neurological disorders (see Arbo et al., 2015).

Using the TSPO ligand Ro5-4864, Barron et al. (2013) assessed the effects of increasing activity of TSPO on neurosteroidogenesis, Aβ accumulation, gliosis and behavioral impairments in male 3xTg-AD mice, investigating both early (7-month old) and late (24-month old) stages of pathology. Ro5-4864 reduced Aβ load and gliosis in CA1 in gonadectomized (GDX) young adults, and also in intact aged mice. Ro5-4864 also increased brain progesterone and testosterone levels in pooled limbic structures of GDX young adults, but surprisingly decreased progesterone levels in aged mice. In addition, Ro5-4864 improved performance on the Y maze task for hippocampal-dependent working memory in GDX young adults and intact aged mice (Barron et al., 2013).

The TSPO ligand Ro5-4864 has also been investigated for potential application in peripheral neuropathy, which was discussed earlier in this review with respect to its improvement following neurosteroid treatment. In male Sprague-Dawley rats given streptozotocin injections to induce diabetic neuropathy, Ro5-4864 increased levels of pregnenolone, progesterone and DHT in the sciatic nerves, while reversing impairments in nerve conduction velocity, thermal threshold, skin innervation density, P0 mRNA levels, and activity of Na^+^/K^+^-ATPase (Giatti et al., 2009). As many of these factors were improved in the same way following treatment with DHT or 3α-diol in a previous study (Calabrese et al., 2014), it seems likely that the effects of Ro5-4864 in this study were due to its regulation of endogenous neurosteroidogenesis.

Like Ro5-4864, the TSPO agonist 4’-Chlorodiazepam (4’CD) has been studied for its potential as a neuroprotective agent using *in vitro* models. In SH-SY5Y cells and organotypic hippocampal cultures from early postnatal Wistar rats, 4’CD has been shown to protect against cell death induced by Aβ40 or Aβ42 (Arbo et al., 2016, 2017). This protection appeared to occur through mechanisms involving prevention of Aβ-induced alterations in Survivin and Bax in SH-SY5Y cells (Arbo et al., 2016), while 4’CD increased expression of superoxide dismutase in organotypic cultures, indicating improved antioxidant activity and potential clearance of oxidative free radicals (Arbo et al., 2017). While these studies did not investigate neurosteroid levels following Aβ or 4’CD treatment, it can be inferred that the protective effects observed in these studies were due to increased steroidogenesis, as many others have demonstrated that protection induced by agonism or over-expression of TSPO can be blocked by inhibitors of neurosteroid synthesis (Santoro et al., 2016; Zhang et al., 2016).

Certain ligands for TSPO are used as anxiolytics in humans, as they increase production of inhibitory neurosteroids like allopregnanolone (Li L. et al., 2017). Etifoxine is one such ligand, which acts as an activator of TSPO and also has neurosteroid-like activity as a positive allosteric modulator of the GABA_A_ receptor (Liere et al., 2017). In a study looking at the impact of acute and chronic effects of etifoxine in male rats, etifoxine acutely increased pregnenolone, progesterone, DHP and allopregnanolone levels in brain tissue and in plasma without impacting testosterone or its metabolites. Concentrations of the neurosteroids peaked 0.5–1 h following injection, and were maintained with daily injections for 15 days (Liere et al., 2017). Through its dual actions of neurosteroid-like activity and stimulation of TSPO activity, etifoxine has been shown to reduce neurological deficits in male mice following intracerebral hemorrhage (ICH) or MCAO-induced hypoxia reperfusion injury (Li H.-D. et al., 2017; Li M. et al., 2017). These improvements were associated with reduced edema, reduced leukocyte infiltration and diminished cell death in the ICH model, reduced infarct volume in the MCAO model, and reduced proinflammatory cytokine production by microglia in both models (Li H.-D. et al., 2017; Li M. et al., 2017).

As TSPO is an important regulator of neurosteroidogenesis, it is interesting to note that increased microglial and astrocytic TSPO immunoreactivity in the hippocampus of AD patients has been reported, with large clusters of TSPO-positive cells localized in close proximity to senile plaques (Cosenza-Nashat et al., 2009). Since TSPO is upregulated in microglia and reactive astrocytes associated with injury (Chechneva and Deng, 2016), this could be a secondary consequence of the cellular damage resulting from Aβ toxicity.

## Modulation of Precursor Actions by Neurosteroids

Aside from their potential role in normalizing pathology-related proteins and intracellular signaling pathways in disease, 3α-hydroxy, 5α-reduced neurosteroids may also act in normal physiological circumstances to dampen cellular responses to their parent hormones, thereby providing an intrinsic regulatory mechanism by which the powerful and potentially harmful actions of steroid hormones may be controlled.

**Figure 3:**
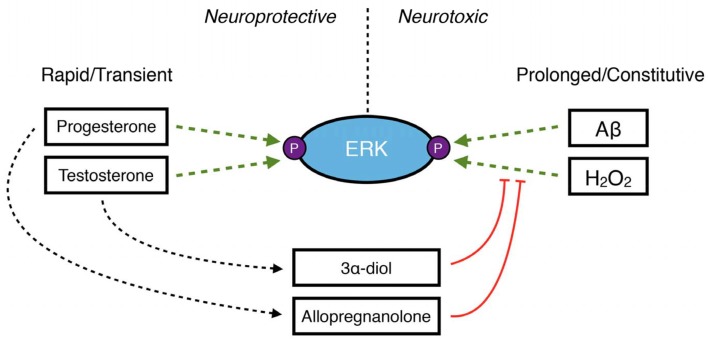
A schematic demonstrating the role of neurosteroids in maintaining the balance of ERK phosphorylation in neuroprotection vs. neurodegeneration. Under normal conditions, the primary gonadal steroid hormones progesterone and testosterone rapidly and transiently increase ERK phosphorylation to protect neurons. However, increased levels of reactive oxygen species like H_2_O_2_ or accumulation of Aβ42 result in prolonged, dysregulated phosphorylation of ERK, which has detrimental downstream consequences in neurons. Under these conditions, the 3α-hydroxy, 5α-reduced neurosteroids 3α-diol and allopregnanolone may inhibit ERK phosphorylation.

Gonadal steroid hormones exert neurotrophic and neuroprotective actions in the brain through several mechanisms, one of which is rapid and transient activation of intracellular signaling pathways leading to regulation of factors that promote neuronal survival (Nguyen et al., 2005; Zhao and Brinton, 2007; Pike et al., 2008). This includes the phosphoinositide 3-kinase/PI3K/Akt/mammalian target of rapamycin (PI3K/Akt/mTOR) pathway, the Ras/Raf/MEK/ERK pathway and the cAMP/PKA pathway, among others (for review see Foradori et al., 2008; Pike et al., 2008; Srivastava et al., 2011; Galea et al., 2017). However, as discussed above, the neurosteroid metabolites 3α-diol and allopregnanolone inhibit some of these same pathways when they become dysregulated in disease and injury (Huang et al., 2016; Mendell et al., 2016, 2018; Figure 3). The same inhibitory mechanisms may limit excessive activation by their precursor hormones—testosterone and progesterone, respectively. In SH-SY5Y human neuroblastoma cells, ERK phosphorylation is rapidly activated by DHT, and this effect is inhibited by co-treatment with 3α-diol (Mendell et al., 2016). As progesterone has been shown to increase ERK phosphorylation in both *in vitro* neuronal models (Singh, 2001; Nilsen and Brinton, 2003) and *in vivo* in the brain (Orr et al., 2012), it is possible that a similar mechanism exists for progesterone metabolites in the regulation of effects driven by their precursor. While this concept presents an intriguing endogenous regulatory system, further work is necessary to determine whether these mechanisms operate in the brain, and whether any sex differences in these effects may exist.

## Neurosteroidogenesis, Biosynthetic Enzymes and Neurosteroid Targets: Sex Differences and Relationship to Neurodegenerative Disease

### Neurosteroidogenesis

The availability of information regarding sex and age differences in neurosteroid levels in humans is limited for many reasons, including technical, logistical and ethical considerations. Furthermore, the influence of 5α-reduced neurosteroid levels on neurodegenerative disease, and the impact of disease progression on neurosteroidogenesis, is largely unknown. For example, AD is among the most widely studied neurodegenerative conditions, with known sex differences in incidence and severity of symptoms (Buckwalter et al., 1993; Henderson and Buckwalter, 1994; Ripich et al., 1995; Xing et al., 2015; Ardekani et al., 2016). However, 5α-reduced metabolites of gonadal steroid hormones have not been thoroughly investigated, though allopregnanolone has been consistently shown to be reduced in individuals with AD (Bernardi et al., 2000; Yang and He, 2001; Marx et al., 2006; Smith et al., 2006; Naylor et al., 2010). Studies in humans based on individual measures make causal inferences problematic: whether decreased allopregnanolone contributed to, or was a result of, disease development cannot be definitively stated.

Studies investigating sex differences in production of 3α-hydroxy, 5α-reduced neurosteroids under physiological conditions are limited. However, an important study by Caruso et al. (2013b) demonstrated that there are sex- and region-dependent differences in neurosteroid levels throughout the CNS and in plasma in young adult Sprague-Dawley rats. Measurements were reported in the cerebrospinal fluid (CSF), hippocampus, cerebral cortex, cerebellum, spinal cord and sciatic nerve—all regions that have been discussed in relation to neurosteroid intervention in models of disease throughout this review. Allopregnanolone levels were modestly higher in plasma and throughout the CNS of females, with the exception of the CSF, where levels were higher in males. The pattern of allopregnanolone levels also matched those of its immediate precursors, progesterone and DHP. In contrast, 3α-diol levels were markedly higher in plasma and in all regions of the CNS investigated in male rats. This pattern also matched those of its precursors, testosterone and DHT (Caruso et al., 2013b). While this study provided important insights into sex differences in levels of 3α-hydroxy, 5α-reduced neurosteroids under normal conditions, more of its kind are necessary.

Though many questions remain unanswered, studies using rodent models have helped to provide important insights into the potential roles for neurosteroids in neurodegenerative disease. In another study by Caruso et al. (2013a), neurosteroid levels were measured in the limbic region of the brain in male 3xTg-AD mice at early (7 months old) and late (24 months old) stages of pathology, compared to age-matched wild-type animals. Pregnenolone, progesterone, testosterone and DHT were all found to decline, while levels of allopregnanolone were unchanged (Caruso et al., 2013a). Interestingly, 3α-diol levels were actually found to increase with age in male mice (Caruso et al., 2013a), which may be particularly important when considered together with reports that male 3xTg-AD mice develop less severe limbic region Aβ pathology and perform better on a task of hippocampal-dependent working memory than age-matched females (Carroll et al., 2010).

Giatti et al. (2010) measured levels of progesterone and testosterone, as well as their 5α-reduced metabolites in the spinal cord, cerebellum, cerebral cortex and plasma in male and female EAE pathogen-free Dark Agouti rats, used as a model of MS. These animals exhibit similar neurological deficiency and pathological severity, though males present with slightly more pronounced inflammation. In all CNS regions, testosterone, DHT and 3α-diol were drastically reduced in male EAE rats, and levels of these neurosteroids ranged from extremely low to undetectable in either control or EAE female rats (Giatti et al., 2010). Progesterone, DHP and allopregnanolone levels were higher in female control rats compared to male controls, but showed a more complex sex- and disease-dependent relationship than testosterone and its metabolites. Progesterone and DHP decreased in both male and female EAE rats in the spinal cord and cerebral cortex, while allopregnanolone actually increased in EAE males and was unaffected in EAE females compared to controls in both brain regions. In the cerebellum, allopregnanolone decreased in EAE females but not males, while progesterone decreased in both male and female EAE rats, and DHP was unaffected (Giatti et al., 2010). This study illustrates the complex nature of alterations to neurosteroidogenesis in disease, in this case using an MS rat model. It is important to note that age was not considered as a factor in this study, which could further compound the interactions between sex- and disease-related effects on neurosteroid levels.

Other studies have investigated changes in neurosteroid levels in rodent models of induced neurological deficits, as opposed to models of disease that occur spontaneously or due to genetic factors. One such study performed by Lopez-Rodriguez et al. (2016) investigated the impact of right orbitofrontal and perirhinal focal lesions in male Swiss-CD1 male mice as a model of TBI on neurosteroid levels in the brain and plasma. The authors reported that progesterone levels were unaffected at time points from 24 h up to 2 weeks following TBI, while testosterone, DHT and 3α-diol exhibited more complex temporal changes. Testosterone levels were significantly increased 2 weeks after injury compared to 24 h, but not significantly different than baseline levels. However, both DHT and 3α-diol were significantly reduced 2 weeks following injury compared to intact controls, while 3α-diol levels were positively correlated with the degree of brain edema, suggesting that a compensatory response may have occurred to increase local neuroprotective activity (Lopez-Rodriguez et al., 2016). Plasma levels of DHT, 3α-diol and 3β-diol were all reduced 2 weeks after TBI, though only plasma DHT and 3α-diol correlated significantly with neurological deficits (Lopez-Rodriguez et al., 2016).

### Neurosteroid Biosynthetic Enzymes and Targets

Aside from neurosteroid levels themselves, measurements of expression and functionality of neurosteroidogenic enzymes, as well as targets for neurosteroid action, are important indicators of potential dysfunction in neurodegenerative disease. In both humans with AD (He et al., 2005; Luchetti et al., 2011a) and mouse models of AD (He et al., 2002; Yang et al., 2014; Porcu et al., 2016), 17β-HSD type 10 (17β-HSD10) is known to increase. As this mitochondrial enzyme functions to oxidize 3α-hydroxy, 5α-reduced neurosteroids, especially in the hippocampus (He et al., 2005), increased expression and/or activity of 17β-HSD10 contributes to decreased neurosteroid levels in AD (Yang et al., 2014; Porcu et al., 2016). Concentrations of the 3α-hydroxy, 5α-reduced neurosteroids are at least partially dependent on what has been referred to as a “dual enzyme molecular switch” (He et al., 2005; Yang et al., 2014; Porcu et al., 2016), involving 17β-HSD10 and 3α-HSD type 3 (3α-HSD3), which is predominantly localized to the endoplasmic reticulum. Theoretically, 3α-hydroxy, 5α-reduced neurosteroid levels would be most affected if the increase in 17β-HSD10 was accompanied by a decrease in 3α-HSD3, or if 5α-reductase expression or activity itself was decreased, thereby decreasing availability of precursors. However, data regarding the activity of these enzymes in neurodegenerative diseases is severely limited, with one study reporting increased expression of 3α-HSD3 in the prefrontal cortex of AD patients with both sexes grouped according to Braak stage (Luchetti et al., 2011a), while others have reported increased 5α-reductase expression in 12-month old APP/PS1 double transgenic AD mice (Porcu et al., 2016). Conversely, 3α-HSD3 and 5α-reductase type 1 expression have both been found to decrease in the white matter of patients with MS and in the hindbrain of young adult EAE mice, though all subjects were pooled and sex differences were not evaluated (Noorbakhsh et al., 2011).

As previously discussed, TSPO is an important regulator of neurosteroid synthesis. Interestingly, TSPO has been found to increase in several neurological and neurodegenerative diseases in humans (Porcu et al., 2016; reviewed in Rupprecht et al., 2010). In AD patients, positron emission tomography (PET) scanning using specific ligands for TSPO have demonstrated increased binding in many cortical areas, indicative of increased TSPO expression (Cagnin et al., 2001; Yasuno et al., 2008, 2012; Venneti et al., 2009; Zimmer et al., 2014). TSPO radioligand binding has also been reported to increase with age and pathology in double transgenic APP/PS1 mice (Venneti et al., 2009). In MS and stroke patients, increased TSPO expression has also been found at the sites of white matter lesions and primary lesions, respectively (Gerhard et al., 2005; Versijpt et al., 2005). Similar findings have been reported in Huntington’s disease (Pavese et al., 2006), ALS (Turner et al., 2004) and Parkinson’s disease (Gerhard et al., 2006). It has been postulated that increased TSPO expression in these various neurodegenerative and neurological conditions could potentially be a compensatory biological response intended to increase local neurosteroid synthesis (Rupprecht et al., 2010), as TSPO levels have been reported to remain elevated during recovery in some disease models, including EAE mice (Agnello et al., 2000; Chen et al., 2004). The findings from these various studies strongly support a mechanism through which endogenous neurosteroid synthesis may increase locally around lesion sites, to limit neurotoxicity. However, it is important to note that sex was not considered as a biological variable in these studies, as male and female human subjects were grouped together in each case.

In addition to biosynthetic enzymes, the receptor targets for 3α-hydroxy, 5α-reduced neurosteroids may themselves be altered in disease states, thereby reducing the potential effectiveness of endogenous neurosteroids. The expression of some known neurosteroid-sensitive GABA_A_ receptor subunits–α1, α2, α4, δ—have been shown to decrease in the prefrontal cortex of AD patients (Luchetti et al., 2011b), concurrent with declines in allopregnanolone levels (Bernardi et al., 2000; Marx et al., 2006; Smith et al., 2006; Naylor et al., 2010). However, the decline in GABA_A_ receptor subunit expression may actually be a consequence of declining neurosteroid levels. Allopregnanolone has been shown to increase hippocampal α4 subunit protein expression in adult female rats (Gulinello et al., 2001; Hsu et al., 2003), and hippocampal synaptic α2 transcript expression in wild-type and progesterone receptor knockout female mice (Reddy et al., 2017). Other novel targets of 3α-hydroxy, 5α-reduced neurosteroids are the PXR and LXR, as discussed above. These proteins are associated with modulation of cholesterol metabolism and homeostasis, which is known to be dysregulated in early and progressive stages of AD (Zelcer et al., 2007). While loss of LXR and PXR was not reported in AD mouse models (Chen et al., 2011), genetic knockout of LXR isoforms exacerbated Aβ pathology in both male and female APP/PS1 double transgenic mice (Zelcer et al., 2007). Allopregnanolone treatment prior to development of Aβ pathology in male 3xTg-AD mice was reported to increase the expression of both LXR and PXR, concurrent with decreased Aβ oligomer accumulation (Chen et al., 2011). These findings provide further evidence that 3α-hydroxy, 5α-reduced neurosteroids, specifically allopregnanolone, may actually promote the expression of their target receptors. Therefore, when levels of these neurosteroids decline in AD, reduced target receptor expression may decline as well. This could indirectly contribute to loss of neuroprotection. For instance, reduced expression or activity of GABA_A_ receptor subunits associated with decreased allopregnanolone in the brain of AD patients could reduce the effectiveness of other neurosteroids that have not been observed to decline, but protect neurons through the same mechanisms. This includes 3α-diol, which may be an important contributing factor to the observed sex differences in the development and progression of AD.

## Conclusions and Limitations

**Figure 4:**
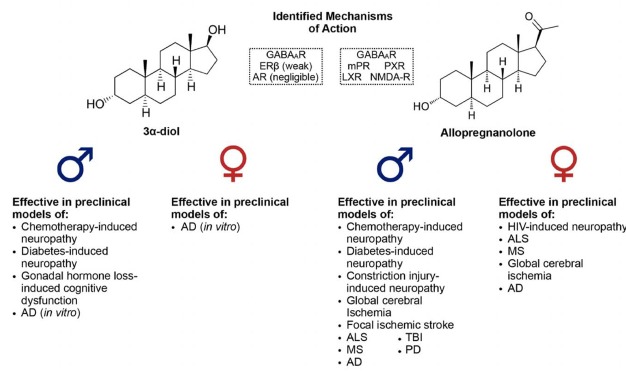
Identified mechanisms of action of 3α-diol and allopregnanolone, along with a list of neurodegenerative/neurological diseases in which these 3α-hydroxy, 5α-reduced neurosteroids have been shown to effectively improve pathology or impairments in preclinical models.

3α-hydroxy, 5α-reduced neurosteroids contribute to the neuroprotective and neurotrophic effects of their precursors, and play important roles in the development—and potential treatment—of neurodegenerative and neurological diseases. While this review has aimed to highlight the sex differences in endogenous production of these neurosteroids and their efficacy for treatment in several rodent models of disease (see Figure 4), there remains a substantial lack of information regarding differences in neurosteroidogenesis throughout life in both humans and other preclinical models, especially in relation to sex as a biological variable, since the majority of studies have been performed in males. This is a vital issue to consider, as it will be a substantial challenge for researchers to fully understand the role of these neurosteroids in sex differences until these questions are answered. The apparent lack of studies concerning both sexes for some of the preclinical disease models discussed should also be considered as a limitation to this review article, and a proposed focus for future studies. Studies incorporating both sexes, investigating age-related changes in neurosteroidogenesis, the onset and progression of disease-related pathology, and potential mechanisms for intervention, are an important priority for future research.

## Author Contributions

AM and NM wrote the manuscript.

## Conflict of Interest Statement

The authors declare that the research was conducted in the absence of any commercial or financial relationships that could be construed as a potential conflict of interest.
